# Metabolic Impact of Adult-Onset, Isolated, Growth Hormone Deficiency (AOiGHD) Due to Destruction of Pituitary Somatotropes

**DOI:** 10.1371/journal.pone.0015767

**Published:** 2011-01-19

**Authors:** Raul M. Luque, Qing Lin, José Córdoba-Chacón, Papasani V. Subbaiah, Thorsten Buch, Ari Waisman, Hugo Vankelecom, Rhonda D. Kineman

**Affiliations:** 1 Research and Development Division, Jesse Brown Veterans Affairs Medical Center, Chicago, Illinois, United States of America; 2 Department of Medicine, Section of Endocrinology, Diabetes and Metabolism, University of Illinois at Chicago, Chicago, Illinois, United States of America; 3 Department of Cell Biology, Physiology and Immunology, University of Córdoba, Instituto Maimónides de Investigación Biomédica de Córdoba (IMIBIC), and CIBER Fisiopatología de la Obesidad y Nutrición (CIBERobn), Córdoba, Spain; 4 Neuroimmunology Division, Institute of Experimental Immunology, Department of Pathology, University Hospital Zürich, Zürich, Switzerland; 5 Institute for Molecular Biology, University Medical Centre, University of Mainz, Mainz, Germany; 6 Laboratory of Tissue Plasticity, Department of Molecular Cell Biology, University of Leuven (K.U.Leuven), Leuven, Belgium; Massachusetts General Hospital, United States of America

## Abstract

Growth hormone (GH) inhibits fat accumulation and promotes protein accretion, therefore the fall in GH observed with weight gain and normal aging may contribute to metabolic dysfunction. To directly test this hypothesis a novel mouse model of adult onset-isolated GH deficiency (AOiGHD) was generated by cross breeding rat GH promoter-driven Cre recombinase mice (Cre) with inducible diphtheria toxin receptor mice (iDTR) and treating adult Cre^+/−^,iDTR^+/−^ offspring with DT to selectively destroy the somatotrope population of the anterior pituitary gland, leading to a reduction in circulating GH and IGF-I levels. DT-treated Cre^−/−^,iDTR^+/−^ mice were used as GH-intact controls. AOiGHD improved whole body insulin sensitivity in both low-fat and high-fat fed mice. Consistent with improved insulin sensitivity, indirect calorimetry revealed AOiGHD mice preferentially utilized carbohydrates for energy metabolism, as compared to GH-intact controls. In high-fat, but not low-fat fed AOiGHD mice, fat mass increased, hepatic lipids decreased and glucose clearance and insulin output were impaired. These results suggest the age-related decline in GH helps to preserve systemic insulin sensitivity, and in the context of moderate caloric intake, prevents the deterioration in metabolic function. However, in the context of excess caloric intake, low GH leads to impaired insulin output, and thereby could contribute to the development of diabetes.

## Introduction

Circulating growth hormone (GH) and insulin-like growth factor I (IGF-I) levels steadily rise after birth, plateau around puberty, then decline thereafter at a rate of ∼14% for every decade of life [1]. GH levels also decline with weight gain, independent of age [2]. Since GH has both lipolytic and anabolic properties [3], it is hypothesized that the decline in GH with age and weight gain is in part responsible for the progression of metabolic disease. Therefore, multiple studies have been conducted to examine the impact of GH replacement in elderly and obese individuals [4]–[6] and off-label use and abuse of GH is becoming more prevalent in healthy adults to enhance body image and athletic performance. However, the true impact of age and weight-related alterations in endogenous GH levels on adult health and disease remains to be clarified, because the bulk of our knowledge is based on studies of 1) short-term GH administration in normal subjects, 2) prolonged GH excess due to GH-producing pituitary tumors, 3) developmental GHD, that might not reflect the consequences of GH decline after sexual maturation and 4) adult onset GHD (AOGHD) due to pituitary surgery or head trauma, which is frequently accompanied by other pituitary defects, making it difficult to determine what changes are due specifically to GH loss. For these reasons, this report describes the development and characterization of a mouse model of adult-onset, isolated GHD (AOiGHD), where experiments were conducted to determine the impact of AOiGHD on whole body insulin sensitivity and glucose tolerance, body composition and circulating and hepatic fat accumulation. Results revealed a partial reduction in endogenous GH levels has both positive and negative effects on metabolic function depending on nutritional status.

## Materials and Methods

### Ethics Statement

This study was carried out in strict accordance with the recommendations in the Guide for the Care and Use of Laboratory Animals of the National Institutes of Health. The protocol was approved by the Institutional Animal Care and Use Committees of Jesse Brown VA Medical Center (Protocol #07-06 and 10-03) and the University of Illinois at Chicago (Protocol #07-036 and 09-246). All surgeries were performed under isoflurane anesthesia and tail vein blood samples were taken after application of anesthetic cream (2.5% lidocaine, 2.5% prolocaine).

### Animals

**Figure 1:**
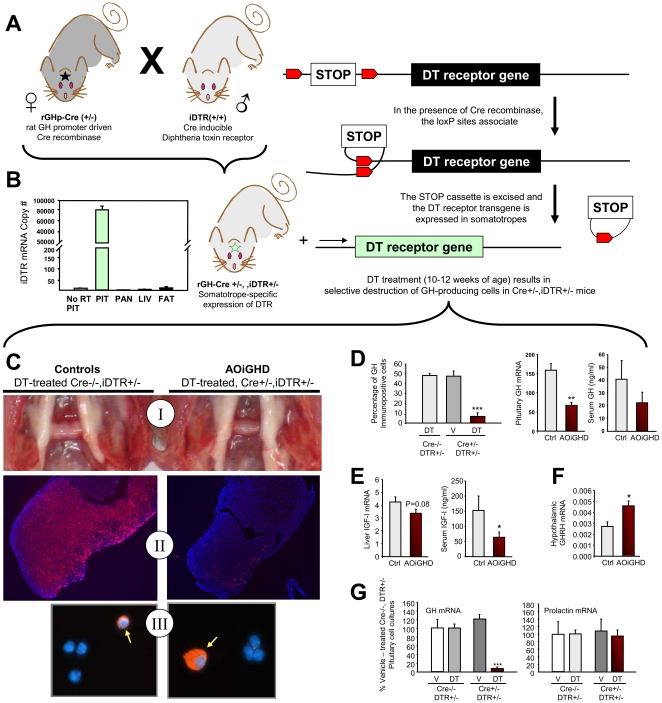
Design and validation of the AOiGHD mouse model. (**A**) rGHp-Cre and iDTR transgenic mice were crossbred to generate mice that express the DTR in the GH-producing cells (somatotropes) of the anterior pituitary gland, (**B**) Analysis of DTR mRNA copy number in pituitary (PIT), pancreas (PAN), liver (LIV) and adipose tissue (FAT) from Cre^+/−^,DTR^+/−^ mice (without DT treatment) reveals that DTR is expressed only in the pituitary (other tissues tested [but not shown] were muscle, spleen, brain, lung, kidney, skin, intestine, adrenal, testis, ovary), “No RT PIT” indicates PCR amplification of total pituitary mRNA which was not reversed transcribed. The tissue specific pattern of DTR mRNA matched that previously reported for Cre recombinase mRNA in rGHp-Cre Tg mice [7]. Cre^+/−^,DTR^+/−^ mice are normal until treated with DT, which results in the destruction of the somatotrope population. (**C**) Gross morphology (I) and GH immunofluorescence of pituitary cross-sections (II) and enzymatically dispersed pituitary cells (III) from DT-treated Cre^−/−^,DTR^+/−^ mice (controls, left panels) and DT-treated Cre^+/−^,DTR^+/−^ mice (AOiGHD, right panels). GH immuno-positive cells appear red and cell nuclei appear blue (II and III). Somatotropes from DT treated Cre^+/−^,DTR^+/−^ mice are enlarged, relative to DT treated controls (III arrows). (**D**) Percentage of GH immuno-positive cells in pituitaries from DT-treated Cre^−/−^,iDTR^+/−^ mice and vehicle (V)- and DT-treated Cre^+/−^,DTR^+/−^ mice (left), and pituitary GH mRNA (middle) and circulating GH (right), in DT-treated control and AOiGHD mice. (**E**) Liver IGF-I mRNA (left) and circulating total IGF-I (right). (**F**) Hypothalamic expression of GHRH. Analysis of circulating hormones and tissue expression analysis were conducted 7 months post DT treatment. Hormone mRNA copy numbers were adjusted by HPRT mRNA copy number to control for total RNA reverse transcribed and the efficiency of the reaction. (**G**) Impact of in vitro vehicle- (V) and DT-treatment (10ng/ml for 24h) on GH and prolactin mRNA levels in primary pituitary cell cultures from adult Cre^−/−^,DTR^+/−^ and Cre^+/−^,DTR^+/−^ mice, where values are the means of 3 independent experiments. Panels C–F represent data collected after 2 months post-DT treatment with n = 8–12 mice/group. Asterisks indicate values that differ from controls, *, p<0.05, ** p<0.01, and *** p<0.001.

A mouse model of AOiGHD was generated by crossbreeding rat GH promoter driven Cre-recombinase (Cre) mice [7] to inducible monkey diphtheria-toxin receptor (iDTR) mice [8], both in a C57BL/6 background (**Fig. 1A**). Mice heterozygous for iDTR, with (Cre^+/−^,iDTR^+/−^) and without (Cre^−/−^,iDTR^+/−^) Cre-recombinase, were treated with DT between 10–12wks of age either by multiple ip injections (4ng/g BW, 2×/day for 10d), or by continuous low dose delivery via osmotic pumps (6ng/h for 7d), where pumps were surgically placed and removed under isoflurane anesthesia. Mice were maintained on a standard rodent chow diet (fat, 17 kcal%; carbohydrate, 56 kcal%; protein 27 kcal% - Formulab Diet, Purina Mills, Inc., Richmond, IN) or fed a low fat diet (LF: fat, 10% kcal%; carbohydrate, 70 kcal%; protein, 20 kcal% - Research Diets, New Brunswick, NJ) starting at 4 weeks of age, where a subset of mice were switched to a high fat diet fat (HF: fat, 45% kcal; carbohydrate, 35 kcal%; protein 20 kcal% - Research Diets), immediately following DT treatment.

### Pituitary phenotype

Pituitary cell cultures or paraffin embedded pituitary sections were processed for immunocytochemistry as previously described [7] using rabbit polyclonal antibodies, directed against rat GH, mouse PRL, human ACTH, rat TSH ß-subunit, or rat LH ß-subunit (Dr. A.F. Parlow, NHPP,NIDDK). In addition, specificity of DT-mediated cell destruction was tested by treating pituitary cell cultures prepared from Cre^−/−^,iDTR^+/−^ and Cre^+/−^,iDTR^+/−^ mice (200,000-cells/well, 3-wells/cultured) with DT (10ng/ml) for 12h. Cells were washed 3×, cultured for an additional 24h and total RNA extracted for assessment of GH and PRL mRNA levels as described below.

### mRNA analysis

Total RNA was extracted from tissues or pituitary cell cultures, reversed transcribed and amplified by quantitative real-time PCR [7]. Primer sequences, Genbank accession numbers and product sizes are provided as supplemental information (**Table-S3**). In studies examining the impact of DT on pituitary GH, liver IGF-I and hypothalamic GHRH mRNA levels, data was adjusted by cyclophilin-A mRNA levels, to control for variations in the amount of RNA used and efficiency of the RT reaction. In studies evaluating the impact of DT on expression of hormones in whole pituitaries taken from LF and HF fed mice, the expression of some housekeeping genes varied with diet, therefore the mRNA copy number for the transcript of interest was adjusted by a normalization factor (NF) calculated from the mRNA copy number of three separate housekeeping genes (glyceraldehyde-3-phosphate dehydrogenase, hypoxanthine ribosyltransferase and cyclophilin-A) using the GeNorm 3.3 program [9].

### Circulating hormones and metabolites

GH, IGF-I, insulin, leptin, corticosterone, testosterone, thyroid hormone (T4) and prolactin levels were assessed using ELISA kits. Triglycerides, NEFA and cholesterol levels were determined using reagents and microtiter plate procedures from WAKO Diagnostics. Blood glucose was assessed by glucometer (OneTouch, SureStep, Johnson & Johnson, Milpitas, CA).

### Liver lipid analysis

Liver was homogenized in PBS containing protease inhibitors and an aliquot extracted according to the method of Bligh and Dyer [10]. Lipids were resuspended in 0.1% Triton-X 100 in PBS and aliquots were assessed for triglycerides. H&E stained liver sections were also compared between groups for the amount of unstained intracellular space within hepatocytes, indicative of lipid accumulation.

### *In vivo* evaluation of metabolic status

Glucose tolerance tests were performed after an overnight fast and insulin tolerance tests were performed under *ad libitum* fed conditions between 0800h–1100h. Blood was collected at t0, for hormone and nutrient measurements, as described above. Assessment of respiratory metabolism (O2 consumption/CO2 production), food and water intake, activity and rearing measurements were performed in house using the PHYSIOCAGE system and METABOLISM analysis software (Panlab Harvard Apparatus, Barcelona, Spain). Whole body composition was assessed by NMR (MiniSpec LF50, Bruker Optics, Manning Park, Billerica MA).

### Statistics

Student's t-tests were used to evaluate the impact of DT on GH-axis function in Cre^+/−^,DTR^+/−^ and Cre^−/−^,DTR^+/−^ mice. Comparison of the effect of genotype and diet on circulating hormones, tissue mRNA transcript levels, response to GTT and ITT was assessed by ANOVA, followed by Newman Keul's post-hoc test. For data collected from the PHYSIOCAGE system, the average day and night VO2, VCO2 and EE and the cumulative food and water intake (24h) and activity levels (day or night) from two consecutive light cycles were compared between genotype, within diet, by student's t-test. RQs collected over a 48h period were pooled for each group and used to calculate the percent relative cumulative frequency (PRCF), as previously described [11] where statistical comparisons were based on the 50^th^ percentile values and curve slopes.

## Results

### Development and characterization of the AOiGHD model

Cre^+/−^,iDTR^+/−^ mice express DTR only in the pituitary (**Fig. 1B**), due to somatotrope-specific, Cre-mediated excision of the floxed STOP cassette 5′ of the iDTR transgene. In the absence of DT, expression of DTR did not impact somatotrope morphology and number (**Fig. 1C–D**), growth, GH/IGF-I output or metabolic function (**Fig.S1**). However, when adult Cre^+/−^,iDTR^+/−^ mice (10–12wks) are treated with DT there is a dramatic reduction in the size of the anterior pituitary gland, compared to DT-treated Cre^−/−^,iDTR^+/−^ controls, where morphology of the DT-treated Cre^−/−^,iDTR^+/−^ controls were identical to that of untreated Cre^−/+^,iDTR^+/−^ (**Fig. 1C**) and WT mice (data not shown). Reduced pituitary size was associated with a reduction in the proportion of GH-producing cells, GH mRNA and circulating GH (**Fig. 1C,II** **and** **Fig. 1D**), as well as a reduction in liver IGF-I mRNA and circulating IGF-I (**Fig. 1E**). However, the DT-mediated decline in GH was not proportional to the ∼90% reduction in somatotrope numbers. This discrepancy may be explained by reduced negative feedback of GH/IGF-I at the level of the hypothalamus and pituitary. This hypothesis is consistent with the fact that the few remaining somatotropes in DT-treated Cre^+/−^,DTR^+/−^ pituitaries are hypertrophied and intensely GH immunopositive (**Fig. 1C,III** **and Fig.S2**), and hypothalamic expression of GHRH is increased (**Fig. 1F**). Nonetheless, these compensatory mechanisms are not sufficient to maintain normal levels of GH/IGF-I.

Since a subpopulation of lactotropes arise from somatotropes during pituitary development [7], some lactotropes could express DTR and be sensitive to DT-mediated destruction. However the destructive impact of DT on lactotropes appears to be minimal since treatment of Cre^+/−^,DTR^+/−^ pituitary cells with DT did not significantly alter prolactin mRNA, but did dramatically reduce GH mRNA levels when compared to DT-treated Cre^−/−^,DTR^+/−^ cultures (**Fig. 1G**). Also, *in vivo* DT treatment did not negatively affect the appearance of the other pituitary cell types (**Fig. S2A)**. To confirm that the functional capacity of the other pituitary cell types was not altered, circulating levels of prolactin, testosterone, T4 and corticosterone were measured in DT-treated Cre^+/−^,iDTR^+/−^ mice and found to not differ from controls (**Table-1**). In addition, the time to conception and litter size of DT-treated Cre^+/−^,DTR^+/−^ male or female mice bred to wildtype-controls, did not differ from that observed using wildtype-C57Bl/6 mice (**Table-S1**). Finally, all of the DT-treated Cre^+/−^,DTR^+/−^ female mice successfully cared for their litters and pup weight at weaning did not differ from control litters. Taken together these results indicate lactotropes, gonadotropes, thyrotropes and corticotropes are not overtly altered by somatotrope destruction, thus confirming DT-treated, Cre^+/−^,DTR^+/−^ adult mice are appropriate models of AOiGHD.

**Table 1:** Circulating levels (ng/ml) of pituitary (regulated) hormones, in AOiGHD and Controls.

	Males*		Females	
	Control	AOiGHD	Control	AOiGHD
**Prolactin**	1.75±0.27	1.6±0.13	43.11±6.8	34.11±3.25
**Testosterone**	7.42±2.78	5.42±1.76	ND	ND
**Thyroid hormone (T4)**	5.42±0.22	5.51±0.13	6.78±0.24	7.64±0.45
**Corticosterone fed**	1.38±0.25	1.41±0.25	2.86±0.85	3.86±0.59
**Corticosterone fasted**	34±2.09	33.8±3.22	33.33±5.7	32.38±4.32

*Values shown for males are from LF- fed mice described in **Fig 3** **(from t0 samples of GTT or ITT), while female values are from chow fed mice. Hormone levels were compared between genotypes from blood samples taken at the same age, from 2–6 months post DT treatment (n = 6–12 samples/measurement).**

### AOiGHD alters metabolic function

**Figure 2:**
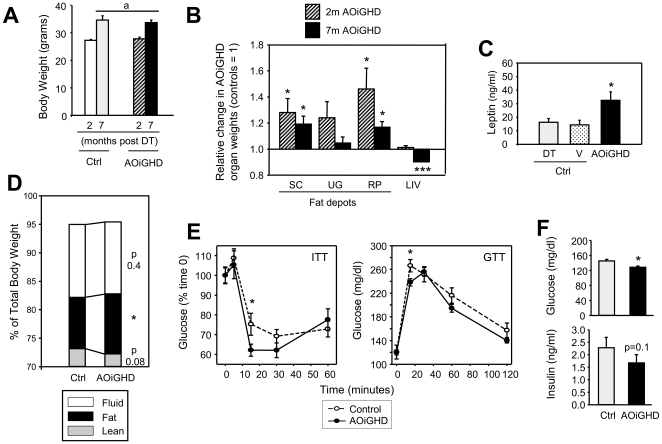
Impact of 2 to 7 months of AOiGHD on metabolic endpoints in male mice fed a standard rodent chow diet. (**A**) Body weights (**B**) Relative fat depot (subcutaneous [SC], urogenital [UG], retroperitoneal [RP]) and liver (LIV) weight, of DT-treated Cre^+/−^,DTR^+/−^ (AOiGHD) mice compared to DT-treated Cre^−/−^,DTR^+/−^ controls set at 1. (**C**) Serum leptin levels measured 2 months post DT treatment in DT-treated Cre^−/−^,DTR^+/−^ and vehicle-treated Cre^+/−^,DTR^+/−^ mice (controls [ctrl]), compared to AOiGHD mice. (**D**) Percent total body weight of free fluid, fat and lean tissue weight measured by whole body NMR 2.5 months post DT treatment, using the MiniSpec LF50 (Bruker Optics, Germany). (**E**) Insulin tolerance tests (ITT, 0.75U/kg ip) and glucose tolerance tests (GTT, 2g/kg ip) conducted 2–3 months post DT treatment. (**F**) Glucose and insulin levels measured in serum samples (trunk blood) 7 month post DT treatment. Values are means +/− SEM, of n = 8–12 mice/group. Asterisks indicate values which significantly differ from controls, * p<0.05, *** P<0.001. a, indicates a significant effect of age (p<0.05), independent of GH status. Data presented in panels A–B and E–F are from AOiGHD and littermate controls, where DT was delivered by multiple ip injections, while data shown in panel D is from a separate set of mice (2.5 months post-DT treatment), where DT was delivered by osmotic minipump as described in Fig. 3. It should be noted that these metabolic endpoints did not differ between Cre^+/−^,DTR^+/−^ and Cre^−/−^,DTR^+/−^ mice not treated with DT, as shown in Fig. S1.

For initial studies, male mice were group housed and provided standard rodent chow (17% total kcal from fat). At 10wk of age, DT was administered by multiple injections, as originally reported [8]. Body weight increased from 2m to 7m post-DT treatment in both AOiGHD and GH-intact controls, but did not differ due to GH status, within age (**Fig. 2A**). However, AOiGHD mice had relatively more fat-mass, compared to controls (**Fig. 2B**), with significant differences in subcutaneous and retroperitoneal depots. Enhanced fat-mass was associated with an increase in circulating leptin (**Fig. 2C**). The increase in fat-mass was confirmed in a separate set of AOiGHD mice, using whole body NMR (p = 0.04, **Fig. 2D**), and this increase was associated with a decrease in lean-mass (p = 0.08). After 7m of AOiGHD, there was a small but highly significant reduction in liver weight (**Fig. 2B**). In contrast, the absolute and relative weight of kidneys, spleen and testis were not significantly altered (data not shown). Despite the increase in fat-mass, AOiGHD mice (2–3m post-DT) displayed a modest improvement in the response to insulin and glucose, with significant differences at 15min post-injection (**Fig. 2E**). The fact that AOiGHD mice also had reduced glucose and insulin levels 7m post-DT (**Fig. 2F**) suggests improved insulin sensitivity persists with age.

**Figure 3:**
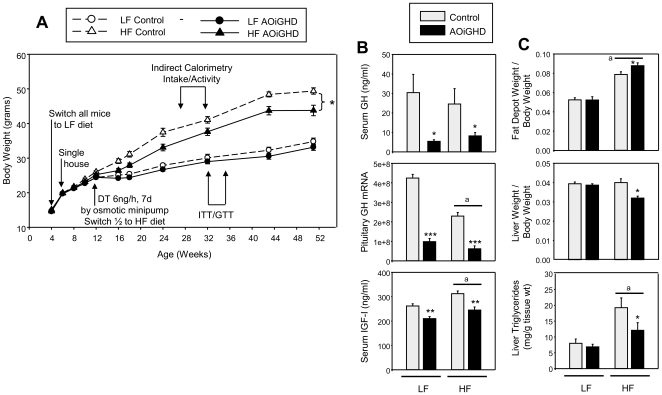
Optimized paradigm to generate AOiGHD mice and compare the impact of a high fat and low fat diet. (**A**) Growth curves, (**B**) Circulating GH and IGF-I levels (from t0 GTT samples, Fig. 3A) and pituitary GH mRNA (copy number/0.05µg total RNA adjusted by a normalization factor of 3 separate housekeeping genes, see methods for details). (**C**) Fat depot (combined subcutaneous, urogenital and retroperitoneal fat pads) and liver weights adjusted by body weight and liver triglyceride content (mg/g tissue weight). Asterisks indicate values which significantly differ from controls, * p<0.05, ** p<0.01, *** P<0.001. “a” indicates significant impact of diet, independent of GH status (p<0.05). Values are means +/− SEM of n = 12–17 mice/group.

In the next series of experiments, modifications were made to the protocol in order to 1) decrease animal to animal variation, 2) limit the nonspecific toxic effects of bolus DT-treatment and 3) better model a “western” diet. Specifically, all experimental mice were generated by breeding female rGHpCre^+/−^ mice to male iDTR^+/+^ mice and litter size was standardized to 6–8 pups/litter 72h after parturition, based on evidence that early nutritional status can dramatically impact adult metabolic and GH-axis function [12]. Mice were weaned at 3wks of age, placed on a low fat (LF, 10% total kcal from fat) diet at 4wks and single housed at 6wks. The rationale to switch all mice to a LF diet at an early age is based on the fact that even with standard chow feeding, C57Bl/6 mice quickly gain fat-mass at variable rates, which may mask effects of AOiGHD. As adults, Cre^+/−^,DTR^+/−^ and Cre^−/−^,iDTR^+/−^ male mice were treated with a low, continuous dose of DT via subcutaneous, osmotic minipump, and half of the mice were switched to a high fat diet (HF, 45% total kcal from fat). The experimental paradigm is illustrated in **Fig. 3A**, along with body weight changes over time.

Circulating GH and IGF-I, and pituitary expression of GH were reduced in DT-treated Cre^+/−^,DTR^+/−^ mice in both diet groups (**Fig. 3B**). In contrast to the suppression of GH/IGF-I in DT-treated Cre^+/−^,DTR^+/−^ mice, expression of the other pituitary hormones (POMC, glycoprotein hormone α-subunit, and ß-subunits of TSH, FSH and LH) were significantly elevated, while there was a small but significant reduction in prolactin mRNA levels in DT-treated Cre^+/−^,DTR^+/−^ mice (**Fig.S2,B**). However, circulating prolactin and testosterone did not differ between control and AOiGHD mice within diet (**Fig.S2,B**). These observations, taken together with our initial findings (**Fig. 1** and **Table-1**), indicate that somatotrope depletion persists with age in DT-treated Cre^+/−^,DTR^+/−^ mice, while other pituitary cell types remain relatively intact and functional. However, it should be noted that independent of GH-status, HF-feeding significantly suppressed pituitary GH mRNA levels (**Fig. 3B**) and testosterone levels (**Fig.S2,B**), while enhancing circulating IGF-I levels (**Fig. 3B**), as previously reported [13]–[15]. Therefore, the metabolic impact of GHD in high-fat mice should be considered in the context of altered testosterone and IGF-I levels.

Body weights of LF-fed AOiGHD and GH-intact control mice did not significantly differ up to 12m of age (**Fig. 3A**). However, body weights of HF-fed AOiGHD mice were significantly less than HF-fed controls. Since mice continue to grow after puberty (albeit at a slower rate), the differences observed in the HF-fed groups may be related to the combined effects of low GH and testosterone in AOiGHD mice, where both hormones are required for optimal skeletal and muscle growth [16]. Relative fat-depot and liver weight (assessed at 12m) did not differ due to GH status in LF-fed mice. In contrast, relative fat-depot weight was increased and liver weight decreased in HF-fed AOiGHD mice compared to controls (**Fig. 3C**), a relationship similar to that observed in younger AOiGHD mice fed a standard chow diet (**Fig. 2B**). The decrease in liver weight in HF-fed AOiGHD mice was associated with decrease in liver triglycerides (**Fig. 3C**), without changes in circulating triglycerides, NEFA or cholesterol (**Table-S2**). Examination of H&E stained liver sections from AOiGHD and control mice fed a standard chow diet revealed less unstained area in AOiGHD hepatocytes, indicative of reduced fat accumulation (**Fig.S3**).

**Figure 4:**
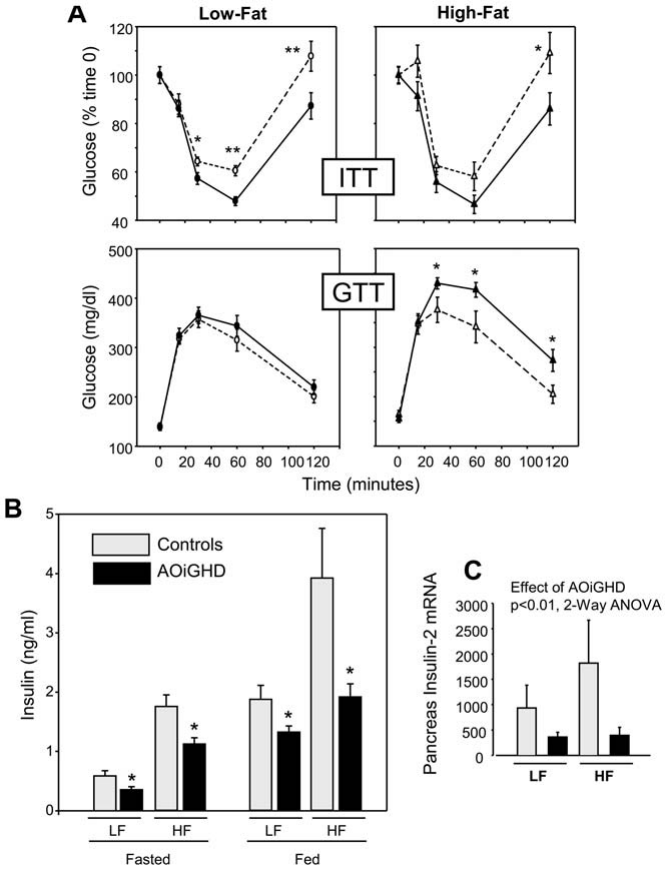
Impact of AOiGHD and diet on glucose homeostasis. (**A**) Insulin tolerance tests (ITT, 1U/kg ip, top panels) and glucose tolerance tests (GTT, 1g/kg ip, bottom panels) in LF (left panels) and HF (right panels) fed AOiGHD (solid lines) and control (dotted lines) mice, performed at 5–6 months post DT treatment. (**B**) Insulin levels under fed and fasted (overnight) conditions, measured from tail vein blood samples collected at t0 in ITT and GTT, respectively. (**C**) Insulin-2 mRNA levels (copy number/0.05 µg total RNA adjusted by a normalization factor of 3 separate housekeeping genes, see methods for details). Asterisks indicate values which significantly differ from controls, * p<0.05, ** p<0.01. Values are means +/− SEM, of n = 8–17 mice/group.

To evaluate the interaction of AOiGHD and diet on glucose homeostasis, ITT and GTT were performed at 5–6m post-DT (**Fig. 4A**). Fed and fasted glucose levels were increased in response to HF diet but were not altered by GH-status (**Table-S2**). The response to GTT did not differ between LF-fed, AOiGHD and control mice, while response to ITT was significantly improved (**Fig. 4A**, and AUC based on absolute glucose levels (mg/dl); controls 13819±635 *vs.* AOiGHD 12021±459, p<0.02) and this was associated with a significant reduction in fed and fasted insulin levels (**Fig. 4B**).

HF-feeding impaired insulin-mediated glucose clearance in both control and AOiGHD mice (AUC, LF-controls 13819±635 *vs.* HF-controls 16391±808 [p<0.05] and LF-AOiGHD 12021±459 *vs.* HF-AOiGHD 14893±829 [p<0.05]). However, in both HF- and LF-fed mice, glucose levels remained significantly lower in AOiGHD mice at the later time points following insulin injection (**Fig. 4A**). Despite these differences in insulin sensitivity, the response to GTT deteriorated in HF-fed AOiGHD mice relative to controls (**Fig. 4A**, AUC HF-controls 36398±2835 vs. HF-AOiGHD 43046±1590, p<0.05). These differences may be attributed to the fact that the compensatory rise in insulin was blunted in HF-fed AOiGHD mice, resulting in a significant difference in insulin levels between AOiGHD and controls, under fasted and fed conditions (**Fig. 4B**). These results suggest that GH/IGF-I may be important in maintaining ß cell function, which is supported by the observation that the level of whole pancreatic INS-2 mRNA, the primary transcript contributing to circulating insulin in mice, was significantly suppressed by AOiGHD (**Fig. 4C**).

**Figure 5:**
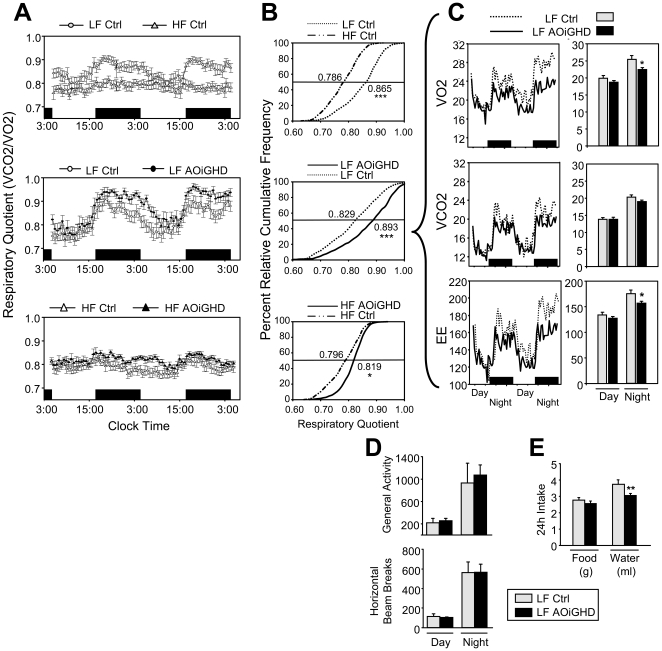
Indirect calorimetry, activity and food/water intake. (**A**) 48h profiles of respiratory quotient (RQ = VCO2 (ml/min/kg^0.75^) / VO2 (ml/min/kg^0.75^) where data was pooled from two separate runs using different mice in each run (n = 10 mice/group total). Comparisons were first made between LF- and HF-fed control mice to verify the accuracy of the gas monitoring system (top panel, 4 months post DT treatment), then AOiGHD mice were compared to controls within diet (LF, 5 months post DT treatment [middle panel] and HF, 5.5 months post DT-treatment [bottom panel]). (**B**) Percent relative cumulative frequency of 48h RQ values was calculated as reported by Riachi et al [11], and 50^th^ percentile RQ values (values shown adjacent to each curves and demarcated by intercepts of the horizontal solid lines) were compared. (**C**) Left panels show the mean 48h profiles and right panels show the day and night averages of VO2, VCO2 and energy expenditure (EE, kcal/day/kg^0.75^) of LF-fed control vs. LF-fed AOiGHD mice conducted in a single run (n = 5 mice/group). (**D**) Impact of AOiGHD on day and night activity levels. (**E**) Impact of AOiGHD on 24h ad libitum food and water intake. Asterisks indicate values that differ from controls, *, p<0.05, ** p<0.01, and *** p<0.001.

To further evaluate the impact of AOiGHD, whole body metabolic function was evaluated by indirect calorimetry and activity level and food/water intake was monitored. The overall respiratory quotient (RQ) of LF-fed AOiGHD was significantly elevated compared to LF-fed controls (**Fig. 5A–B**, middle panels; note right shift in the percent relative cumulative frequency distribution [PRCF] of RQs, 50th percentile LF-controls RQ 0.829 vs. LF-AOiGHD RQ 0.893, p<0.001) and this difference was more pronounced during the absorptive state (night-time feeding). As illustrated in **Fig. 5C**, the increase in RQ in LF-fed AOiGHD mice was associated with a significant decrease in nocturnal VO_2_ and energy expenditure (EE), without significant changes in VCO_2_. These changes were not associated with significant alterations in mean activity levels (**Fig. 5D**) or food intake (**Fig. 5E**). Interestingly, LF-fed, AOiGHD mice drank less water than controls, which may be related to changes in body composition or due to changes in cardiovascular/renal function (**Fig. 5E**). HF-feeding reduced the RQ in both control (50^th^ percentile RQ 0.796) and AOiGHD (50^th^ percentile RQ 0.819) mice (**Fig. 5A–B**, bottom panels) and blunted the dynamic diurnal rhythms in RQ typically observed in LF-fed mice, as illustrated by the steeper slopes of PRCF curves in HF- vs. LF-fed mice. Despite this reduction, the overall RQ remained significantly higher (p<0.05) in AOiGHD mice, as compared to controls. Independent of GH-status, HF feeding also decreased activity, increased the amount of calories consumed, but had no effect on the mass (grams) of food consumed, while decreasing water intake (**Fig.S4**), as previously reported by other laboratories [17], [18].

## Discussion

### AOiGHD improves whole body insulin sensitivity

These studies reveal AOiGHD is associated with both positive and negative effects on metabolic function depending on age and diet. Common across age and diet was the observation that AOiGHD improved whole body insulin sensitivity. These findings are consistent with the improved insulin sensitivity reported in developmental GHD and GH insensitive (GHRKO) mice [19]–[21], but are in contrast to the majority of clinical studies showing adults with GHD are insulin resistant [22]–[24]. These apparent species differences may be more related to diet, lifestyle, environmental factors and/or etiology and specificity of GHD, based on the finding that a rural Brazilian family with isolated GHD due to an inactivating mutation in the GHRH-R, were also found to be more insulin sensitive, as compared to their normal counterparts [25], [26]. In addition adult patients with Laron syndrome (inactivating mutations in the GH receptor) remained relatively insulin sensitive (calculated by HOMA-IR), despite an obese phenotype [27]. Finally, adult patients with both GHD and type-I diabetes were shown to require less insulin to normalize glucose and therefore suffered from more frequent hypoglycemic events, and these features were normalized after GH therapy [28]. Improved insulin signaling in the context of isolated GHD is consistent with the well accepted anti-insulin actions of GH following an acute bolus injection in normal and GHD adults [29], and as observed following chronic GH elevation in patients with GH producing pituitary tumors [30], as well as in mice overexpressing GH [21].

The exact mechanism by which GH reduces insulin sensitivity remains to be clarified. However, since GH promotes fat breakdown, it is hypothesized that FFA play a role in GH-mediated insulin resistance. This hypothesis is supported by the observation that the anti-insulin actions of short-term GH treatment are blunted by the anti-lipolytic agent, acipimox [31]. However, in the current study circulating NEFA and triglyceride levels were not altered by GH status, indicating GH-mediated changes in insulin sensitivity can occur, independent of changes in circulating lipids. Our results are consistent with reports showing GH-mediated insulin resistance occurs in liver-specific IGF-I knockout (LID) mice, which have a 75% reduction in circulating IGF-I levels leading to a 3–4 fold increase in GH, with no significant alteration in circulating FFA levels [32]. Also LID mice crossed with GHR antagonist transgenic mice were shown to have improved insulin sensitivity despite elevated FFA levels [32]. Finally, a lipid-independent effect of GH on insulin signaling is suggested by a clinical study showing GH-treatment of GHD, type I diabetics suppressed insulin sensitivity in the presence or absence of heparin infusion [33]. *In vitro* studies also support a direct inhibitory effect of GH on insulin signaling in adipocytes [21], [34].

The observation that AOiGHD mice exhibited an increase in the RQ, without significant alterations in food intake and activity level, indicates carbohydrates are being preferentially utilized for cellular metabolism, consistent with improved insulin sensitivity. A negative relationship between GH and carbohydrate utilization has also been observed in GH-treated, healthy or GHD subjects [35], [36]. Of note, the difference in RQ between AOiGHD and GH-intact mice was most prominent during the evening hours when mice are actively feeding, while mean values for each group steadily converged throughout the day, a time when mice are sleeping (voluntary ‘fast’). Although the rise in GH observed with fasting, which occurs in mice [37] as well as humans, is thought to contribute to a shift in nutrient availability and utilization, the diurnal pattern of RQ in AOiGHD indicates a prolonged reduction in circulating GH levels can also have a profound impact on whole body metabolism in the absorptive state. The limited impact of GHD in the post-absorptive state may be related to the dramatic diurnal patterns in adrenal function reported in mice, where glucocorticoids levels peak at the end of the light phase [38], which may mask any impact of GHD on peripheral insulin sensitivity.

### AOiGHD impairs diet-induced compensatory insulin output

In order for glucose tolerance to remain constant when whole body insulin sensitivity changes, a reciprocal alteration in insulin output has to occur, which is indeed the case in LF-fed AOiGHD mice which show improved insulin sensitivity and low insulin levels under both fed and fasted conditions. However, HF-fed AOiGHD exhibit impaired glucose clearance, despite improved insulin sensitivity, where circulating insulin levels and whole pancreatic INS-2 transcript remained inappropriately low, compared to GH-intact controls. These observations strongly suggest GH/IGF-I is important in maintaining ß-cell function in the adult, a role only previously recognized in mouse models of developmental global and pancreatic GH insensitivity [20], [39], [40]. A direct stimulatory effect of GH on ß-cell function is further supported by the observations that islets do express GHR [41], [42] and GH stimulates ß–cell proliferation and insulin release in normal human islet cultures [43] and protects against inflammatory cytokine-induced apoptosis in ß-cell lines, where these actions require STAT5 activation [44]. There are also isolated clinical studies suggesting GH may support ß-cell function. Specifically, GHD patients display impaired 1^st^ phase insulin release during a hyperglycemic clamp [45] and 30 months of low dose GH replacement improved insulin output in response to oral GTT 3-fold compared to the pre-GH response [46]. Consistent with these observations, non-diabetic acromegalics were shown to have an exaggerated insulin response to glucose stimulation [47]. Admittedly, in clinical trials it is difficult to determine if GH-associated changes in insulin output are direct or due to GH-mediated changes in insulin sensitivity, however these observations coupled with our current findings showing AOiGHD have lower circulating insulin levels in both fed and fasted conditions and reduced pancreatic expression of INS-2, independent of diet, support a role for GH in maintaining adult ß-cell function. It remains to be explored whether this effect is due to changes in ß-cell number and/or amount of insulin produced/cell and if the impact of AOiGHD is mediated by a direct or indirect mechanisms, perhaps via reduction in systemic or local production of IGF-I.

### AOiGHD increases fat depot weight but decreases liver weight and TG content

As observed in humans with AOGHD [2], [45], AOiGHD mice have relatively more fat, compared to GH-intact controls. However these differences were only observed in mice fed a standard chow or HF diet, but not in mice provided a LF diet. Interestingly, an opposite effect of AOiGHD was observed on liver weight and TG content of HF-fed mice, suggesting when calories are in excess they are preferentially stored in adipose tissue reserves, reducing accumulation in the liver, a shift that may be explained at least in part by enhanced insulin sensitivity. Although further studies are required to determine if AOiGHD differentially impacts tissue specific changes in insulin sensitivity, the fact that glucose levels remained significantly lower in LF and HF-fed AOiGHD mice up to 2 hours post insulin injection, suggest hepatic glucose output is decreased in AOiGHD mice, which would be consistent with an improvement in hepatic insulin sensitivity.

The reduction in hepatic TG content in HF-fed AOiGHD mice at first appears in conflict with the reports showing hepatosteatosis develops in mice with liver-specific knockout of STAT5b [48], a key mediator of hepatic GH actions, and in mice with liver-specific knockout of GHR [49]. In the liver-specific GHR knockout mice, lipid accumulation was associated with a decrease in TG secretion [49]. However, it should be noted that liver-specific knockout of GH signaling is associated with low circulating IGF-I leading to a compensatory rise in GH which in turn serves to breakdown fat, elevating circulating FFA levels and reducing whole body insulin sensitivity and glucose clearance. Therefore differences in hepatic accumulation of lipids in AOiGHD mice, as compared with hepatic GH resistance mice, may in fact be indirectly mediated by changes in lipid flux and hepatic insulin signaling. This hypothesis is supported by the observation that bGH transgenic mice are also insulin resistant and show reduced hepatic TG secretion [50]. In addition, it should be noted that developmental hepatic GH resistance (unlike the AOiGHD model) may impact normal development of the liver, where GH is critical for normal hepatocyte proliferation [48]. Therefore it could be argued that in the hepatic GH resistant mouse models there may be a disproportionate influx of lipids relative to hepatic mass.

In summary, this report describes the development and characterization of a unique mouse model of AOiGHD, where circulating GH levels were selectively reduced but not eliminated. As summarized in Table 2, these initial investigations reveal that even partial GHD has a dramatic impact on metabolic function resulting in improved whole body insulin sensitivity independent of diet. With excess caloric intake, AOiGHD leads to an increase in adipose tissue mass, reduced hepatic lipid accumulation, but deterioration of glucose clearance rates, associated with impaired insulin output. From these initial observations we might speculate that the age-related decline in GH may indeed help maintain healthy metabolic function if nutrient intake is restricted. However, GHD may contribute to the development of diabetes in diet-induced obese subjects by limiting insulin output.

**Table 2:** Metabolic changes observed in AOiGHD mice.

		Level of Caloric Intake*	
	Low	Moderate	High
**Body composition**			
Body Weight (g)	**−**	**−**	**↓**
Lean Mass (NMR)	nd	**↓**	nd
Fat Mass (NMR)	nd	**↑**	nd
Fat Depot Weight (g)	**−**	**↑**	**↑**
**Circulating Nutrients (fed/fasted)**			
Triglycerides	**−/−**	nd	**−/−**
Cholesterol	**−/−**	nd	**−/−**
Free Fatty Acids	**−/−**	nd	**−/−**
Glucose	**−/−**	**↓/**nd	**−/−**
**Liver**			
Weight (g)	**−**	**↓**	**↓**
TG content	**−**	**↓**	**↓**
IGF-I (ng/ml)	**↓**	**↓**	**↓**
IGF-I mRNA	**↓**	**↓**	**↓**
**Glucose Homeostasis**			
Respiratory Quotient	**↑**	nd	**↑**
Insulin Sensitivity (ITT)	**↑**	**↑**	**↑**
Insulin fed/fasted (ng/ml)	**↓/↓**	**↓/**nd	**↓/↓**
Pancreatic Insulin mRNA	**↓**	nd	**↓**
Glucose Clearance (GTT)	**−**	**−**	**↓**

*, Low (low-fat diet, 10% calories from fat), Moderate (standard chow diet, 17% calories from fat), High (high-fat diet, 45% calories from fat).

Consistent across diets, male AOiGHD mice are more insulin sensitive, with lower circulating levels of IGF-I and insulin. As caloric intake increases adipose mass increases and liver weight decreases, where the later is associated with reduced triglyceride content (TG). Despite, improved insulin sensitivity, glucose clearance deteriorates in high-fat fed AOiGHD mice. It is hypothesized that this is due to inappropriate pancreatic insulin production, which remains to be tested. Symbols indicate increases (↑), decreases (↓), or no change (−) in AOiGHD mice, relative to GH-intact controls. nd, not determined; NMR, whole body nuclear magnetic resonance; ITT, insulin tolerance test; GTT, glucose tolerance test.

## Supporting Information

Figure S1 Confirmation that the differences observed in AOiGHD (DT-treated Cre^+/−^,DTR^+/−^) and control (DT-treated Cre^−/−^,DTR^+/−^) mice were due to GH deficiency and not to genotype. (A) Growth curves, (B) circulating GH, IGF-I, glucose and insulin, (C) response to insulin tolerance tests (ITT, 1U/kg ip), (D) 48h respiratory quotient (RQ) profiles, as assessed by indirect calorimetry. Male mice were provided a standard rodent chow diet (17% kcal from fat) and were NOT treated with DT. (E) Tissue weight adjusted by body weight, at 10 months of age. There were no difference between genotype in all endpoints examined, where each test was performed at an age similar to those shown for DT-treated mice fed a standard rodent chow diet. n = 8–10 mice/genotype.
(PDF)

Figure S2 Impact of DT treatment on the appearance of pituitary cell types (A, top micrographs), pituitary hormone and receptor mRNA levels and circulating hormones levels (B, lower graphs) in Cre^+/−^,DTR^+/−^ (AOiGHD) mice, compared to Cre^−/−^,DTR^+/−^ (control) mice. (A) Micrographs - Immunocytochemistry for GH, prolactin (PRL), ß-subunit of thyroid stimulating hormone (TSH), ß-subunit of luteinizing hormone (LH) and adrenocorticotropic hormone (ACTH) performed 2 months post DT treatment, in chow fed mice. AOiGHD mice have fewer GH-immunopositive cells which are more intensely stained, while the appearance of the other pituitary cell types were not diminished and in fact appear more concentrated; a situation that is expected if somatotropes, which normally represent 50% of the pituitary cell population, were removed by DT treatment. (B) Graphics – In samples taken from high-fat (HF) and low-fat (LF) fed mice at 12 months of age, somatotrope specific transcripts (GH, GH releasing hormone receptor [GHRH-R] and ghrelin receptor [GHS-R]) were reduced in AOiGHD mice, while PRL mRNA levels were only modestly reduced, but did not lead to a reduction in circulating PRL levels, compared to controls. In contrast, transcripts associated with thyrotropes, gonadotropes and corticotropes were increased, but this did not alter associated circulating hormones (also refer to Table 1 in text). When interpreting the changes in the pituitary mRNA levels, it would be anticipated that the relative expression of hormones produced by non-somatotropes would double if DT-mediated destruction was limited to the majority of somatotropes, which normally make up 50% of all cells in the male pituitary gland. Indeed this was the case for ACTH, LH and TSH mRNAs. However, PRL mRNA levels were the same in LF and modestly reduced in HF conditions in AOiGHD mice compared to controls. This may indicate some lactotropes were destroyed by DT-treatment. An alternative (or additional) explanation is that the reduced levels of IGF-I are responsible for reduced PRL mRNA levels, where IGF-I has been shown to be required for maximal PRL expression. The reduced IGF-I may in fact be a dominant player in reduced PRL mRNA in AOiGHD since DT did not directly suppress PRL mRNA levels in vitro, while having a profound impact on GH mRNA levels, as shown in Figure 1G, in the main text. Asterisks indicate a significant difference between controls and AOiGHD, within diet (p<0.05), as assessed by 2-way ANOVA, followed by Newman Keul's post-hoc tests for group comparisons. Also, HF feeding did have a significant inhibitory impact on GH, GHRH-R, GHS-R and circulating testosterone levels, independent of GH status (a, p<0.05).
(PDF)

Figure S3 Hemotoxylin∶Eosin stained paraffin embedded liver sections from AOiGHD and GH-intact controls. Mice were fed a standard rodent chow diet (17% kcal from fat) and tissues collected at 10 months of age (7 months after DT treatment). In AOiGHD hepatocytes, there is less open (unstained) area, as compared to GH-intact controls, consistent with reduced hepatic triglyceride levels in high-fat fed AOiGHD mice (shown in Fig. 3 in main body of the text).
(PDF)

Figure S4 Comparison of food and water intake (A), kcal consumed (B) and activity levels of control mice (GH-intact) fed either a low fat (LF) or high fat (HF) diet. *, p<0.05 and ** p<0.01.
(PDF)

Table S1 Fertility endpoints in AOiGHD mice, compared to wildtype (WT) mice in a C57Bl/6J background *All 5 female AOiGHD mice successfully cared for their litters until weaning (d21). † Similar data was obtained from C57Bl/6J females housed at the University of Cordoba, Spain (days to conception 6.1±2.4; pups/litter 5.1±0.4 - Raul M. Luque and Jose Cordoba, unpublished data). Jackson Laboratories report C57Bl/6J mice are “good” breeders with 3–7 pups/litter http://jaxmice.jax.org/strain/000664.html
(PDF)

Table S2 Impact of AOiGHD and diet on circulating metabolites. Fed and fasted samples were taken at t0 of ITT and GTT, respectively, where GTT was performed 3 weeks after ITT, on control and AOiGHD mice fed a low-fat (LF) or high-fat (HF) diet. Given the age differences between fed and fasted sampling, the impact of GH-status and diet were made within feeding status (*ie.* fed or fasted), using 2-way ANOVA, followed by Newman's Keuls' test for post-hoc comparisons. GH-status, within diet, did not alter the endpoints measured, however, high fat feeding increased circulating glucose and cholesterol levels as compared to LF-fed controls, independent of GH-status, under both fed and fasted conditions (*, p<0.05).
(PDF)

Table S3 PCR primer sets, positions relative Genbank sequence provided and products sizes.
(PDF)
