# Supplementary material for: Metabolic Impact of Adult-Onset, Isolated, Growth Hormone Deficiency (AOiGHD) Due to Destruction of Pituitary Somatotropes
Source: PLoS One. 2011 Jan 19;6(1):e15767. doi: 10.1371/journal.pone.0015767 (PMC3023710; doi:10.1371/journal.pone.0015767)
Supplement: Figure S1 — Confirmation that the differences observed in AOiGHD (DT-treated Cre+/−,DTR+/−) and control (DT-treated Cre−/−,DTR+/−) mice were due to GH deficiency and not to genotype. (A) Growth curves, (B) circulating GH, IGF-I, glucose and insulin, (C) response to insulin tolerance tests (ITT, 1U/kg ip), (D) 48h respiratory quotient (RQ) profiles, as assessed by indirect calorimetry. Male mice were provided a standard rodent chow diet (17% kcal from fat) and were NOT treated with DT. (E) Tissue weight adjusted by body weight, at 10 months of age. There were no difference between genotype in all endpoints examined, where each test was performed at an age similar to those shown for DT-treated mice fed a standard rodent chow diet. n = 8–10 mice/genotype. (PDF) [file pone.0015767.s001.pdf]

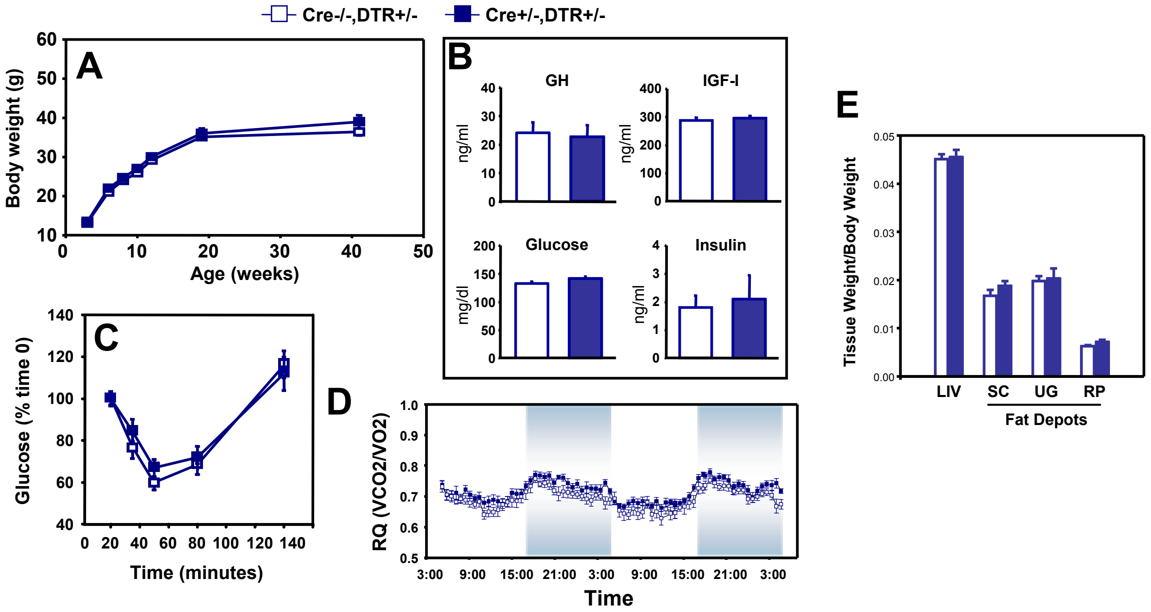

Figure S1

Confirmation that the differences observed in AOiGHD (DT-treated Cre+/-,DTR+/-) and control (DT-treated Cre-/-,DTR+/-) mice were due to GH deficiency and not to genotype. **(A)** Growth curves, **(B)** circulating GH, IGF-I, glucose and insulin, **(C)** response to insulin tolerance tests (ITT, 1U/kg ip), **(D)** 48h respiratory quotient (RQ) profiles, as assessed by indirect calorimetry. Male mice were provided a standard rodent chow diet (17% kcal from fat) and were NOT treated with DT. **(E)** Tissue weight adjusted by body weight, at 10 months of age. There were no difference between genotype in all endpoints examined, where each test was performed at an age similar to those shown for DT-treated mice fed a standard rodent chow diet. n=8-10 mice/genotype.
