# Supplementary material for: Metabolic Impact of Adult-Onset, Isolated, Growth Hormone Deficiency (AOiGHD) Due to Destruction of Pituitary Somatotropes
Source: PLoS One. 2011 Jan 19;6(1):e15767. doi: 10.1371/journal.pone.0015767 (PMC3023710; doi:10.1371/journal.pone.0015767)
Supplement: Figure S2 — Impact of DT treatment on the appearance of pituitary cell types (A, top micrographs), pituitary hormone and receptor mRNA levels and circulating hormones levels (B, lower graphs) in Cre+/−,DTR+/− (AOiGHD) mice, compared to Cre−/−,DTR+/− (control) mice. (A) Micrographs - Immunocytochemistry for GH, prolactin (PRL), ß-subunit of thyroid stimulating hormone (TSH), ß-subunit of luteinizing hormone (LH) and adrenocorticotropic hormone (ACTH) performed 2 months post DT treatment, in chow fed mice. AOiGHD mice have fewer GH-immunopositive cells which are more intensely stained, while the appearance of the other pituitary cell types were not diminished and in fact appear more concentrated; a situation that is expected if somatotropes, which normally represent 50% of the pituitary cell population, were removed by DT treatment. (B) Graphics – In samples taken from high-fat (HF) and low-fat (LF) fed mice at 12 months of age, somatotrope specific transcripts (GH, GH releasing hormone receptor [GHRH-R] and ghrelin receptor [GHS-R]) were reduced in AOiGHD mice, while PRL mRNA levels were only modestly reduced, but did not lead to a reduction in circulating PRL levels, compared to controls. In contrast, transcripts associated with thyrotropes, gonadotropes and corticotropes were increased, but this did not alter associated circulating hormones (also refer to Table 1 in text). When interpreting the changes in the pituitary mRNA levels, it would be anticipated that the relative expression of hormones produced by non-somatotropes would double if DT-mediated destruction was limited to the majority of somatotropes, which normally make up 50% of all cells in the male pituitary gland. Indeed this was the case for ACTH, LH and TSH mRNAs. However, PRL mRNA levels were the same in LF and modestly reduced in HF conditions in AOiGHD mice compared to controls. This may indicate some lactotropes were destroyed by DT-treatment. An alternative (or additional) explanation is that the reduced leve [file pone.0015767.s002.pdf]

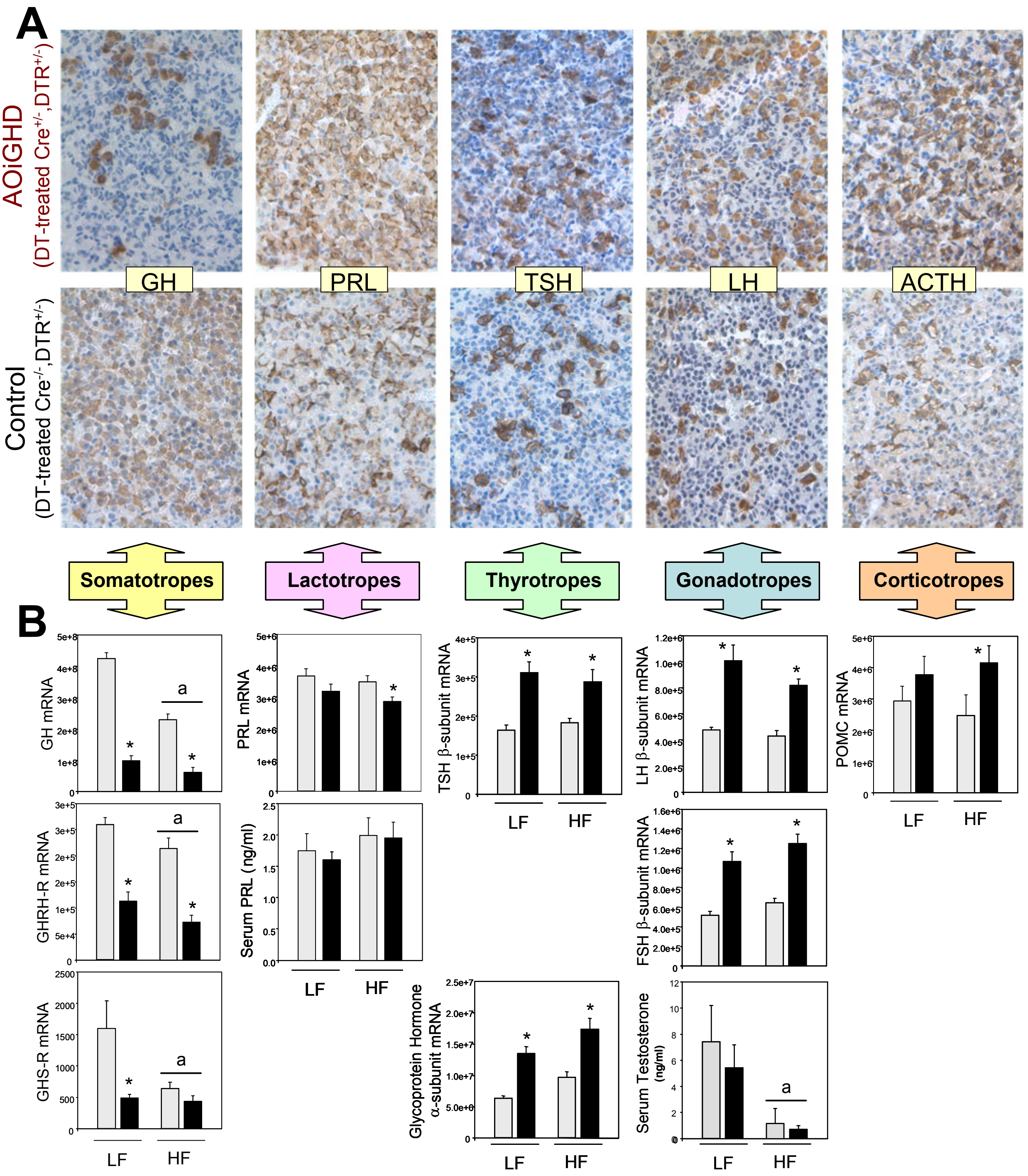

Figure S2

Impact of DT treatment on the appearance of pituitary cell types (A, top micrographs), pituitary hormone and receptor mRNA levels and circulating hormones levels (B, lower graphs) in Cre+/-,DTR+/- (AOiGHD) mice, compared to Cre-/-,DTR+/- (control) mice. **(A)** Micrographs - Immunocytochemistry for GH, prolactin (PRL), β-subunit of thyroid stimulating hormone (TSH), β-subunit of luteinizing hormone (LH) and adrenocorticotropic hormone (ACTH) performed 2 months post DT treatment, in chow fed mice. AOiGHD mice have fewer GH-immunopositive cells which are more intensely stained, while the appearance of the other pituitary cell types were not diminished and in fact appear more concentrated; a situation that is expected if somatotropes, which normally represent 50% of the pituitary cell population, were removed by DT treatment. **(B)** Graphics – In samples taken from high-fat (HF) and low-fat (LF) fed mice at 12 months of age, somatotrope specific transcripts (GH, GH releasing hormone receptor [GHRH-R] and ghrelin receptor [GHS-R]) were reduced in AOiGHD mice, while PRL mRNA levels were only modestly reduced, but did not lead to a reduction in circulating PRL levels, compared to controls. In contrast, transcripts associated with thyrotropes, gonadotropes and corticotropes were increased, but this did not alter associated circulating hormones (also refer to Table 1 in text). When interpreting the changes in the pituitary mRNA levels, it would be anticipated that the relative expression of hormones produced by non-somatotropes would double if DT-mediated destruction was limited to the majority of somatotropes, which normally make up 50% of all cells in the male pituitary gland. Indeed this was the case for ACTH, LH and TSH mRNAs. However, PRL mRNA levels were the same in LF and modestly reduced in HF conditions in AOiGHD mice compared to controls. This may indicate some lactotropes were destroyed by DT-treatment. An alternative (or additional) explanation is that the reduced levels of IGF-I are responsible for reduced PRL mRNA levels, where IGF-I has been shown to be required for maximal PRL expression. The reduced IGF-I may in fact be a dominant player in reduced PRL mRNA in AOiGHD since DT did not directly suppress PRL mRNA levels *in vitro*, while having a profound impact on GH mRNA levels, as shown in Figure 1G, in the main text. Asterisks indicate a significant difference between controls and AOiGHD, within diet ($p<0.05$), as assessed by 2-way ANOVA, followed by Newman Keul's post-hoc tests for group comparisons. Also, HF feeding did have a significant inhibitory impact on GH, GHRH-R, GHS-R and circulating testosterone levels, independent of GH status (a, $p<0.05$).
