# Supplementary material for: Metabolic Impact of Adult-Onset, Isolated, Growth Hormone Deficiency (AOiGHD) Due to Destruction of Pituitary Somatotropes
Source: PLoS One. 2011 Jan 19;6(1):e15767. doi: 10.1371/journal.pone.0015767 (PMC3023710; doi:10.1371/journal.pone.0015767)
Supplement: Figure S3 — Hemotoxylin∶Eosin stained paraffin embedded liver sections from AOiGHD and GH-intact controls. Mice were fed a standard rodent chow diet (17% kcal from fat) and tissues collected at 10 months of age (7 months after DT treatment). In AOiGHD hepatocytes, there is less open (unstained) area, as compared to GH-intact controls, consistent with reduced hepatic triglyceride levels in high-fat fed AOiGHD mice (shown in Fig. 3 in main body of the text). (PDF) [file pone.0015767.s003.pdf]

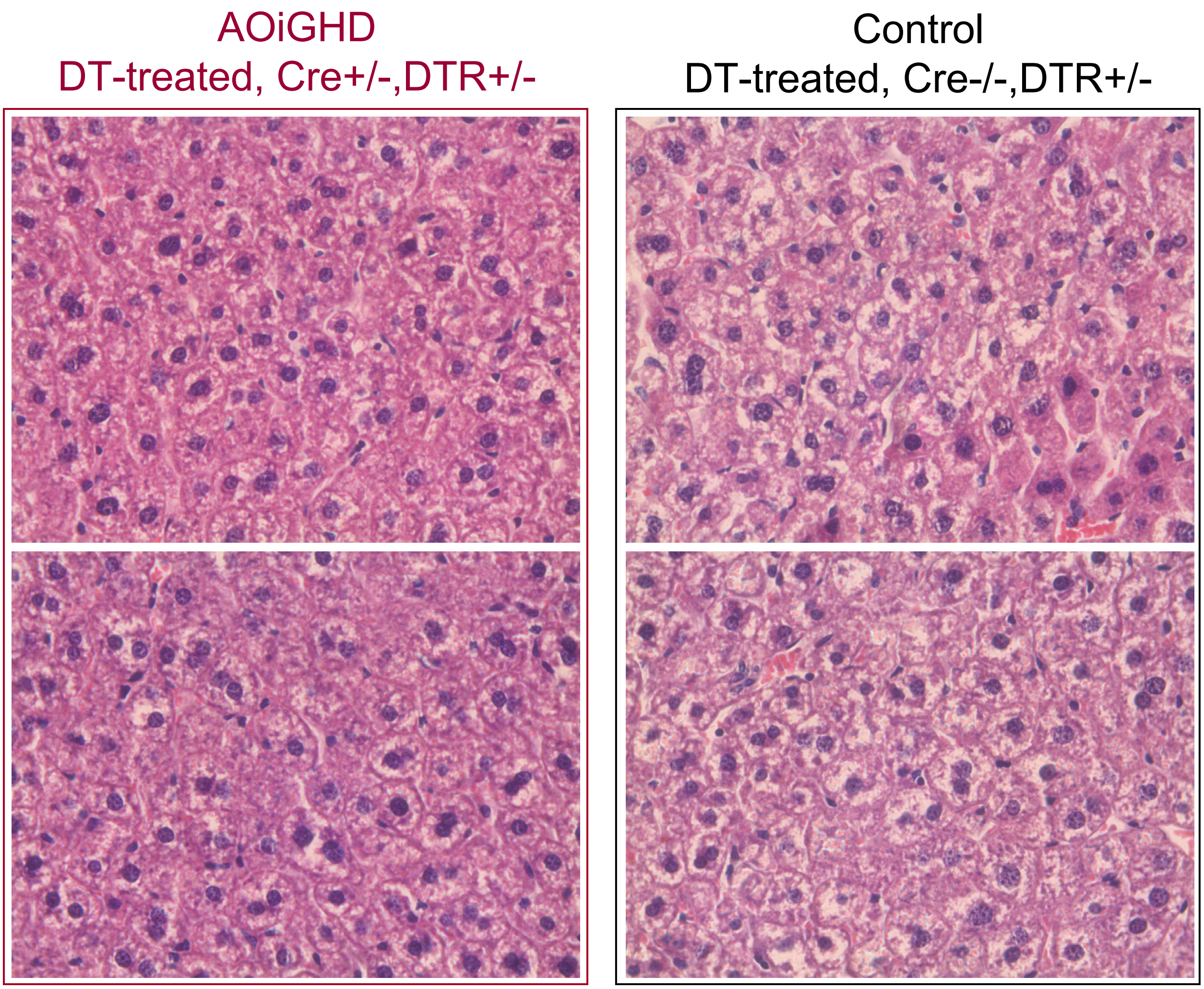


**Figure S3**

Hemotoxylin:Eosin stained paraffin embedded liver sections from AOiGHD and GH-intact controls. Mice were fed a standard rodent chow diet (17% kcal from fat) and tissues collected at 10 months of age (7 months after DT treatment). Within AOiGHD hepatocytes there is less open (unstained) area, as compared to GH-intact controls, indicative of less lipid accumulation. This is consistent with reduced hepatic triglyceride levels in high-fat fed AOiGHD mice (shown in Fig. 3 in main body of the text).
