# Supplementary material for: Metabolic Impact of Adult-Onset, Isolated, Growth Hormone Deficiency (AOiGHD) Due to Destruction of Pituitary Somatotropes
Source: PLoS One. 2011 Jan 19;6(1):e15767. doi: 10.1371/journal.pone.0015767 (PMC3023710; doi:10.1371/journal.pone.0015767)
Supplement: Figure S4 — Comparison of food and water intake (A), kcal consumed (B) and activity levels of control mice (GH-intact) fed either a low fat (LF) or high fat (HF) diet. *, p<0.05 and ** p<0.01. (PDF) [file pone.0015767.s004.pdf]

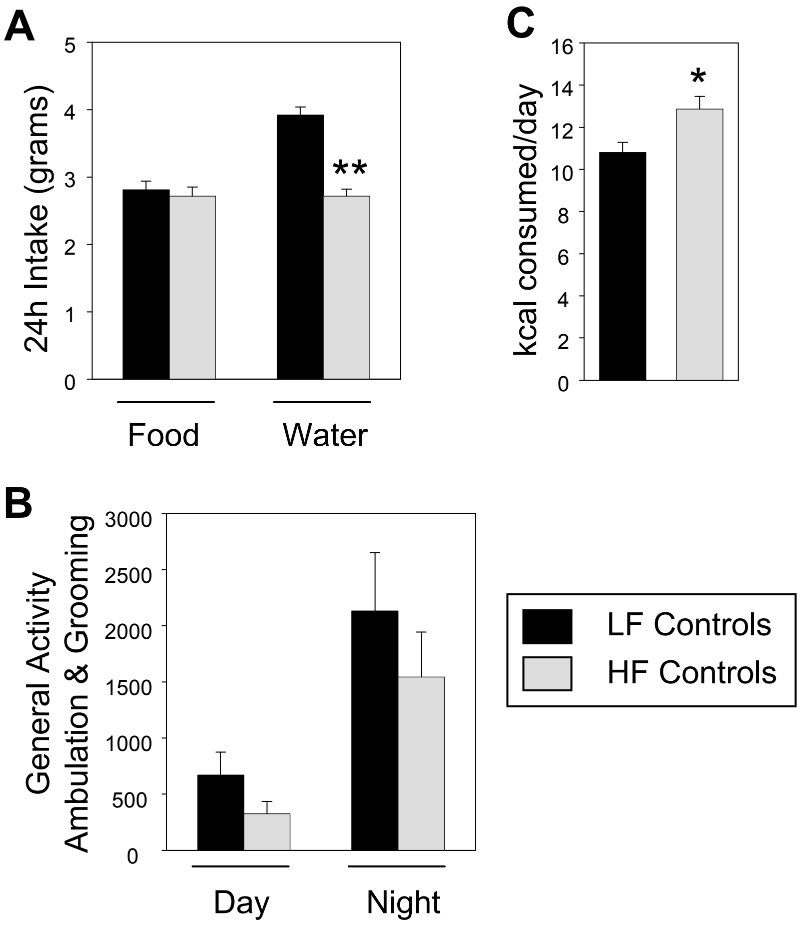

Figure S4

Comparison of food and water intake (A), kcal consumed (B) and activity levels of control mice (GH-intact) fed either a low fat (LF) or high fat (HF) diet. *, p<0.05 and ** p<0.01.
