# Supplementary material for: Metabolic Impact of Adult-Onset, Isolated, Growth Hormone Deficiency (AOiGHD) Due to Destruction of Pituitary Somatotropes
Source: PLoS One. 2011 Jan 19;6(1):e15767. doi: 10.1371/journal.pone.0015767 (PMC3023710; doi:10.1371/journal.pone.0015767)
Supplement: Table S1 — Fertility endpoints in AOiGHD mice, compared to wildtype (WT) mice in a C57Bl/6J background *All 5 female AOiGHD mice successfully cared for their litters until weaning (d21). † Similar data was obtained from C57Bl/6J females housed at the University of Cordoba, Spain (days to conception 6.1±2.4; pups/litter 5.1±0.4 - Raul M. Luque and Jose Cordoba, unpublished data). Jackson Laboratories report C57Bl/6J mice are “good” breeders with 3–7 pups/litter http://jaxmice.jax.org/strain/000664.html (PDF) [file pone.0015767.s005.pdf]

Table S1. Fertility endpoints in AOiGHD mice, compared to wildtype (WT) mice in a C57Bl/6J background.

Breeding Pairs				
Female	Male	# of Pairs Tested	Days to Conception	Pups / Litter
AOiGHD*	WT	5	4.4 ± 0.8	8.2 ± 0.7
WT	AOiGHD	5	6.2 ± 2.7	7.4 ± 0.3
WT†	WT (in house)	10	4.7 ± 1.1	7.0 ± 0.4
WT (Jackson)	WT (Jackson)			3-7

**All 5 female AOiGHD mice successfully cared for their litters until weaning (d21)*

† Similar data was obtained from C57Bl/6J females housed at the University of Cordoba, Spain (days to conception 6.1±2.4; pups/litter 5.1±0.4 - Raul M. Luque and Jose Cordoba, unpublished data).

Jackson Laboratories report C57Bl/6J mice are “good” breeders with 3-7 pups/litter http://jaxmice.jax.org/strain/000664.html
