# Supplementary material for: Metabolic Impact of Adult-Onset, Isolated, Growth Hormone Deficiency (AOiGHD) Due to Destruction of Pituitary Somatotropes
Source: PLoS One. 2011 Jan 19;6(1):e15767. doi: 10.1371/journal.pone.0015767 (PMC3023710; doi:10.1371/journal.pone.0015767)
Supplement: Table S2 — Impact of AOiGHD and diet on circulating metabolites. Fed and fasted samples were taken at t0 of ITT and GTT, respectively, where GTT was performed 3 weeks after ITT, on control and AOiGHD mice fed a low-fat (LF) or high-fat (HF) diet. Given the age differences between fed and fasted sampling, the impact of GH-status and diet were made within feeding status (ie. fed or fasted), using 2-way ANOVA, followed by Newman's Keuls' test for post-hoc comparisons. GH-status, within diet, did not alter the endpoints measured, however, high fat feeding increased circulating glucose and cholesterol levels as compared to LF-fed controls, independent of GH-status, under both fed and fasted conditions (*, p<0.05). (PDF) [file pone.0015767.s006.pdf]

Table S2 - Impact of AOiGHD and Diet on Circulating Metabolites

		LF		HF	
		Control	AOiGHD	Control	AOiGHD
Glucose (mg/dl)	Fed	145 ± 5	149 ± 5	170 ± 6*	182 ± 6*
	Fast	139 ± 7	136 ± 6	163 ± 9*	155 ± 7*
NEFA (mEq/L)	Fed	0.42 ±0.03	0.40 ± 0.02	0.45 ± 0.02	0.44 ± 0.02
	Fast	0.64 ± 0.06	0.58 ±0.05	0.67 ± 0.03	0.61 ± 0.03
TG (mg/dl)	Fed	34 ± 3	33 ± 2	33 ± 3	36 ± 2
	Fast	29 ± 2	26 ± 1	22 ± 3	23 ± 2
Cholesterol (mg/dl)	Fed	120 ± 3	115 ± 4	171 ± 9*	169 ± 5*
	Fast	115 ± 6	112 ± 4	155 ± 9*	151 ± 6*

Fed and fasted samples were taken at t0 of ITT and GTT, respectively, where GTT was performed 3 weeks after ITT, on control and AOiGHD mice fed a low-fat (LF) or high-fat (HF) diet. Given the age differences between fed and fasted sampling, the impact of GH-status and diet were made within feeding status (*ie* fed or fasted), using 2-way ANOVA, followed by Newman's Keul's test for pos-hoc comparisons. GH-status, within diet, did not alter the endpoints measured, however, high fat feeding increased circulating glucose and cholesterol levels as compared to LF-fed controls, independent of GH-status, under both fed and fasted conditions (*, $p<0.05$).
