# Supplementary material for: Metabolic Impact of Adult-Onset, Isolated, Growth Hormone Deficiency (AOiGHD) Due to Destruction of Pituitary Somatotropes
Source: PLoS One. 2011 Jan 19;6(1):e15767. doi: 10.1371/journal.pone.0015767 (PMC3023710; doi:10.1371/journal.pone.0015767)
Supplement: Table S3 — PCR primer sets, positions relative Genbank sequence provided and products sizes. (PDF) [file pone.0015767.s007.pdf]

Table S3 - PCR primer sets, positions relative Genbank sequence provided and products sizes

	Genbank Accession #	Primer Sequence	Nucleotide Position	Product Size
**CRE**	X03453.1	**Sense:** CGTACTGACGGTGGGAGAAT<br>**Antisense:** CCCGGCAAAACAGGTAGTTA	**Sn** 1043<br>**As** 1208	166
**DTR**	M93012.1	**Sense:** CATCCACGGAGAATGCAAA<br>**Antisense:** ACCACAGCCAGGATAGTTGTATG	**Sn** 406<br>**As** 555	150
**GHRH-R**	NM_001003685.1	**Sense:** ACCCGTATCCTCTGCTTGCT<br>**Antisense:** AGGTGTTGTTGGTCCCCTCT	**Sn** 45<br>**As** 177	133
**GHS-R**	NM_177330.3	**Sense:** TCAGGGACCAGAACCACAAA<br>**Antisense:** CCAGCAGAGGATGAAAGCAA	**Sn** 1002<br>**As** 1072	71
**GHRH**	NM_010285.2	**Sense:** TGCCATCTTCACCACCAAC<br>**Antisense:** TCATCTGCTTGTCCTCTGTCC	**Sn** 203<br>**As** 360	158
**GH**	NM_008117.2	**Sense:** CCTCAGCAGGATTTTCACCA<br>**Antisense:** CTTGAGGATCTGCCCAACAC	**Sn** 412<br>**As** 553	142
**PRL**	NM_011164.1	**Sense:** GGCCATCTTGGAGAAGTGTG<br>**Antisense:** ACAGATTGGCAGAGGCTGAA	**Sn** 14<br>**As** 153	140
**TSH β-subunit**	NM_009432.1	**Sense:** CTCCGTGCTTTTTGCTCTTG<br>**Antisense:** TTGCCATTGATATCCCGTGT	**Sn** 177<br>**As** 332	156
**LH β-subunit**	NM_008497.2	**Sense:** TGTCCTAGCATGGTCCGAGT<br>**Antisense:** AGGAAAGGAGACTATGGGGTCTA	**Sn** 179<br>**As** 316	138
**FSH β-subunit**	NM_008045.2	**Sense:** AGTTGATCCAGCTTTGCATCTT<br>**Antisense:** GCCAGGCAATCTTACGGTCT	**Sn** 70<br>**As** 314	245
**α-subunit**	NM_009889.2	**Sense:** CTAGGAGCCCCCATCTACCA<br>**Antisense:** CACTCTGGCATTTCCCATTAC	**Sn** 242<br>**As** 409	168
**POMC**	NM_008895.3	**Sense:** GAGGCCTTTCCCCTAGAGTT<br>**Antisense:** CACCGTAACGCTTGTCCTT	**Sn** 615<br>**As** 768	154
**IGF-I**	NM_010512.3	**Sense:** TCGTCTTCACACCTCTTCTACCT<br>**Antisense:** ACTCATCCACAATGCCTGTCT	**Sn** 31<br>**As** 232	202
**INS-2**	NM_008387.3	**Sense:** TCAAAAACCATCAGCAAGCA<br>**Antisense:** ACCAGGTGGGAACCACAAA	**Sn** 23<br>**As** 180	158
**Cyclophilin A**	NM_008907.1	**Sense:** TGGTCTTTGGGAAGGTGAAAG<br>**Antisense:** TGTCCACAGTCGGAAATGGT	**Sn** 421<br>**As** 529	109
**GAPDH**	XM_001473623.1	**Sense:** ATGGCCTTCCGTGTTCCTAC<br>**Antisense:** GCCTGCTTCACCACCTTCTT	**Sn** 757<br>**As** 860	104
**β-Actin**	NM_007393.2	**Sense:** CTGGGACGACATGGAGAAGA<br>**Antisense:** ACCAGAGGCATACAGGGACA	**Sn** 313<br>**As** 517	205
